# Preclinical Analysis of Sex-Specific Differences in the Angiogenic and Inflammatory Tissue Response to Surgical Sutures

**DOI:** 10.3390/jfb17050233

**Published:** 2026-05-07

**Authors:** Selina Wrublewsky, Jan Weigl, Caroline Bickelmann, Matthias W. Laschke

**Affiliations:** 1 Institute for Clinical and Experimental Surgery, Saarland University, PharmaScienceHub (PSH), 66421 Homburg, Germanycaroline.bickelmann@uks.eu (C.B.); matthias.laschke@uks.eu (M.W.L.); 2 Center for Gender Specific Biology and Medicine (CGBM), Saarland University, 66421 Homburg, Germany

**Keywords:** polypropylene, suture, foreign body response, inflammation, vascularization, sex

## Abstract

Surgical sutures are widely used biomaterials in clinical practice. Like all other biomaterials, they induce a foreign body response after implantation that involves inflammation and angiogenesis. Although it is well known that these processes differ in males and females, sex-specific differences in the tissue response to sutures have not been investigated so far. To do this in the present study, polypropylene sutures were implanted into the dorsal skinfold chamber and subcutaneous flank tissue of male and female mice to assess their acute and chronic effects on the local tissue microenvironment using intravital fluorescence microscopy and immunohistochemistry over 14 and 28 days, respectively. Microhemodynamic parameters and the numbers of rolling and adherent leukocytes in venules next to the implants were comparable in male and female mice. Immunohistochemical analyses on day 14 revealed a stronger neutrophilic (myeloperoxidase (MPO)^+^ cells: 526 ± 29 mm^−2^) and macrophage (CD86^+^ cells: 188 ± 21 mm^−2^; CD163^+^ cells: 269 ± 25 mm^−2^) response, as well as reduced T-cell activation (CD3^+^ cells: 31 ± 4 mm^−2^) in females when compared to males (MPO^+^ cells: 221 ± 25 mm^−2^; CD86^+^ cells: 120 ± 15 mm^−2^; CD163^+^ cells: 101 ± 19 mm^−2^; CD3^+^ cells: 62 ± 13 mm^−2^), while microvessel density and collagen deposition in the forming granulation tissue around the implants did not differ between sexes. In the flank model, there were no detectable sex-specific differences in the chronic foreign body response. These findings demonstrate that polypropylene sutures provoke a stronger early activation of the innate immune system in females, whereas the chronic foreign body response to the implants is comparable in both sexes.

## 1. Introduction

Surgical sutures belong to the most commonly applied biomaterials in clinical practice [1]. Like all other implanted biomaterials, they induce a foreign body response, which is a highly dynamic and sequential process involving the stimulation of immune cells and the development of new microvessels [2,3]. This foreign body response is typically initiated by an acute inflammatory phase characterized by the rapid recruitment of neutrophilic granulocytes and monocytes differentiating into macrophages. These cells orchestrate the subsequent transition into chronic inflammation, which is associated with the formation of granulation tissue, angiogenesis, and extracellular matrix deposition, reflecting the integration of the biomaterial into the host tissue [4,5,6]. The severity of a foreign body response varies depending on material-specific properties, such as the quantity, type and structural configuration of the implanted biomaterial [7].

It is well known that there are sex-specific differences in the number and activity of immune cell subtypes, which may affect the progression of a foreign body response. For instance, females exhibit neutrophilic granulocytes and macrophages with enhanced phagocytic capacity, resulting in faster clearance of debris in the tissue surrounding implanted materials when compared to males [8,9]. Moreover, sex-dependent variations in blood vessel formation and extracellular matrix deposition have been reported [10,11,12,13,14]. In fact, the density of newly formed blood vessels in aortic valves is higher in males [12], whereas extracellular matrix deposition is lower when compared to females [14].

These observations suggest that the biological response to implanted biomaterials may differ between males and females. However, despite the widespread clinical use of surgical sutures, potential sex-specific differences in the foreign body response to these materials have not been systematically investigated so far, even though they may be of crucial importance for prognosis and the risks associated with tissue regeneration. To address this issue in the present study, we analyzed the sex-specific response to non-degradable polypropylene sutures, which are widely applied in cardiovascular, general, plastic, and orthopedic surgery [15,16,17]. These sutures were first implanted into dorsal skinfold chambers of male and female mice to assess the acute angiogenic and inflammatory host tissue response using repeated intravital fluorescence microscopy and immunohistochemistry. In an additional set of experiments, polypropylene sutures were implanted into the subcutaneous flank tissue of male and female mice for the immunohistochemical analysis of the chronic foreign body response after 4 weeks.

## 2. Materials and Methods

### 2.1. Materials

Polypropylene sutures (Prolene^TM^; size: 0-0) were from Ethicon (Norderstedt, Germany). Fluorescein isothiocyanate (FITC)-labeled dextran 150,000, rhodamine 6G, and Hoechst 33342 were from Sigma-Aldrich (Taufkirchen, Germany). Hematoxylin was from Morphisto (Offenbach am Main, Germany). Ketamine (Ketabel^®^) was from Bela-Pharm GmbH & Co. KG (Vechta, Germany). Xylazine (Rompun^®^) was from Bayer (Leverkusen, Germany). Carprofen (Rimadyl^®^) was from Zoetis Deutschland GmbH (Berlin, Germany).

### 2.2. Antibodies

The anti-CD31 antibody (DIA310) was from Dianova (Hamburg, Germany). The anti-myeloperoxidase (MPO) (ab9535), anti-CD3 (ab16669) and anti-collagen (COL) I (ab270993) antibodies were from Abcam (Cambridge, UK). The anti-CD86 antibody (19589) was from Cell Signaling (Danvers, MA, USA). The anti-CD163 (16646) and anti-COL III (22734) antibodies were from Proteintech (Rosemont, IL, USA). The anti-rat IgG Alexa Fluor 555 antibody (A21434) was from Thermo Fisher Scientific (Karlsruhe, Germany). The peroxidase-labeled anti-rabbit antibody (NIF 824) was from GE Healthcare (Freiburg, Germany).

### 2.3. Animals

Male and female BALB/c wildtype mice (Janvier Labs, Le Genest-Saint-Isle, France) with a body weight of 22–26 g were randomly assigned to the dorsal skinfold chamber (male: n = 8; female: n = 8) and flank model (male: n = 8; female: n = 8). The animals were maintained in a conventional animal facility (Institute for Clinical and Experimental Surgery, Saarland University, Homburg, Germany) on a 12/12 h day/night cycle and received water and standard pellet chow (ssniff Spezialdiäten GmbH, Soest, Germany) ad libitum.

This study was conducted in line with the German legislation on the protection of animals and the National Institutes of Health (NIH) Guide for the Care and Use of Laboratory Animals (Institute of Laboratory Animal Resources, National Research Council, Washington, DC, USA). The experiments were approved by the local authorities (permission number: 03-2024; State Office for Consumer Protection, Saarbrücken, Germany).

### 2.4. Dorsal Skinfold Chamber Model

**Figure 1:**
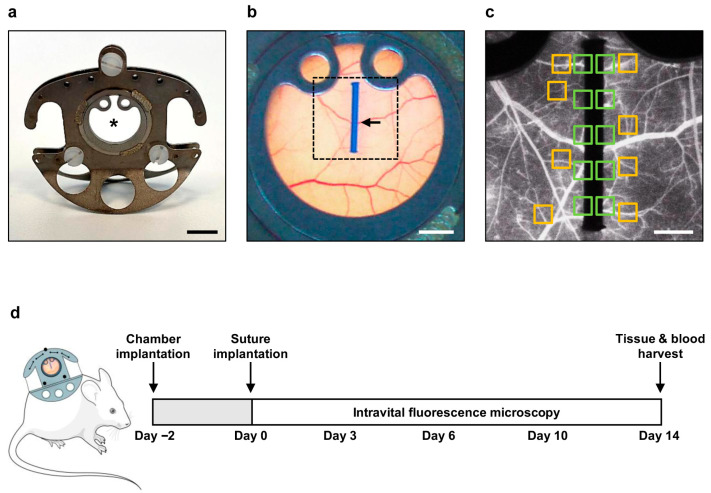
Dorsal skinfold chamber model. (**a**) Image of a dorsal skinfold chamber in mice. The observation window is marked by an asterisk. Scale bar: 5 mm. (**b**) Overview of the observation window in (**a**) after implantation of a polypropylene suture (arrow) on day 0. Image area from (**c**) is marked by broken lines. Scale bar: 2 mm. (**c**) Intravital fluorescent microscopic image after the implantation of a polypropylene suture into the dorsal skinfold chamber on day 0. The intravital fluorescent microscopic analyses included assessment of vascularization in 10 regions of interest (ROIs; green) and leukocyte-endothelial cell interactions in 8 postcapillary and collecting venules (orange). Scale bar: 700 µm. (**d**) Experimental setting. Dorsal skinfold chambers were implanted into male and female mice on day −2, followed by the implantation of a polypropylene suture on day 0. Intravital fluorescence microscopy was performed on days 0, 3, 6, 10 and 14 after implantation. The tissue of the observation window and the blood of the mice were harvested on day 14 after microscopy for immunohistochemistry and blood count, respectively.

The animals were anesthetized by intraperitoneal (i.p.) injection of ketamine (100 mg/kg body weight) and xylazine (12 mg/kg body weight). For analgesia, the animals were subcutaneously injected with carprofen (10 mg/kg body weight). A dorsal skinfold chamber (Figure 1a) was prepared [18]. For this purpose, two symmetrical titanium frames were sutured onto the dorsal skinfold, resulting in the doubling of the skin into two layers. One layer of cutis, subcutis and retractor muscle was excised in a circular area with a diameter of ~15 mm. This area was covered by a cover slip and fixed by a snap ring. Thereafter, the animals could recover for 48 h.

Thereafter, the mice were anesthetized again, the cover glass was removed, and the tissue was carefully flushed with saline. Then, a polypropylene suture with a length of 4 mm was placed into the center of the observation window, which was then closed with a new cover slip (Figure 1b).

### 2.5. Intravital Fluorescence Microscopy

The anesthetized mice with dorsal skinfold chambers received a retrobulbary intravenous injection of 0.05 mL FITC-labeled dextran (5%) for visualization of the plasma, as well as 0.05 mL rhodamine 6G (2%), for in situ leukocyte staining on days 0 (day of suture implantation), 3, 6, 10 and 14. The dorsal skinfold chamber was placed under a fluorescence microscope (Zeiss, Oberkochen, Germany) with a blue (excitation wavelength: 450–490 nm; emission wavelength: >515 nm) and green (excitation wavelength: 530–560 nm; emission wavelength: >585 nm) filter block and the microscopic images were recorded for off-line measurements using CapImage (version 8.5; Zeintl, Heidelberg, Germany).

The functional microvessel density (cm/cm^2^), i.e., the length of all red blood cell (RBC)-perfused blood vessels per ROI, was analyzed in 10 ROIs of the implanted polypropylene suture (Figure 1c). For each examination time point, the mean value from these measurements was calculated for each animal. Leukocyte-endothelial cell interactions were evaluated in 8 postcapillary and collecting venules next to the implant (Figure 1c) by assessing the number of rolling leukocytes (min^−1^) and adherent leukocytes (mm^−2^), as previously described in detail [18]. In addition, the diameter (µm), centerline RBC velocity (µm/s), volumetric blood flow (pL/s), and wall shear rate (s^−1^) of these venules were measured [19]. Again, for each examination time point, the mean value from these measurements was calculated for each animal.

After the last microscopy on day 14, the blood of the anesthetized mice was collected for blood counts (VetScan^®^ HM5; Abaxis, Griesheim, Germany) and the dorsal skinfold chamber tissue, including the implanted suture was excised and fixed for 24 h in 4% paraformaldehyde (PFA) (Figure 1d).

### 2.6. Subcutaneous Flank Model

For operative analgesia, the mice were subcutaneously injected with 10 mg/kg carprofen 30 min before initiation of anesthesia. The animals were anesthetized with isoflurane (3% (*v*/*v*) for induction and 1–2% (*v*/*v*) for maintenance). The flanks of the animals were shaved and depilated. Using a 20G peripheral venous catheter, a subcutaneous tunnel (length: 30 mm) was created caudally to cranially on both sides of the flank. Thereafter, a polypropylene suture (length: 20 mm) was pushed through this tunnel using the catheter needle. In the last step, the needle was removed.

On day 28, the mice were anesthetized by an i.p. injection of ketamine (100 mg/kg body weight) and xylazine (12 mg/kg body weight) and subsequently euthanized by cervical dislocation. Thereafter, the flank tissue, including the implanted suture was excised and fixed for 24 h in 4% PFA.

### 2.7. Immunohistochemistry

The fixed tissues were embedded in paraffin for the cutting of 3 μm thick sections. These sections were stained with the indicated primary and secondary antibodies. Cell nuclei were labeled with Hoechst 33342 for fluorescence microscopy and with hematoxylin for bright-field microscopy. The sections were analyzed by fluorescence and bright-field microscopy (BX60F; Olympus, Hamburg, Germany).

The density (mm^−2^) of CD31^+^ microvessels was evaluated in the granulation tissue surrounding the implanted sutures by dividing the total number of vessels by the granulation tissue area. The density (mm^−2^) of MPO^+^ granulocytes, CD3^+^ lymphocytes, CD86^+^ (M1) macrophages, and C163^+^ (M2) macrophages was evaluated in the granulation tissue surrounding the implanted sutures by dividing the total number of positively stained cells by the granulation tissue area. Additionally, the total COL I and COL III ratio, relative to normal skin, was determined [20]. FIJI software (NIH) was used for assessment of positively stained cells and measurement of areas. For each staining, one section per mouse was analyzed.

### 2.8. Statistical Analysis

All data analyses were conducted in a blinded manner to minimize potential bias. The statistical analysis was performed by means of GraphPad Prism software 11 (Dotmatics, Boston, MA, USA). Following the 3R principle in animal testing, the number of animals per group was reduced to a minimum while guaranteeing sufficient statistical power (>0.8) to detect biologically meaningful differences. Due to low imaging quality, a few measurements in individual animals were not included in the data sets, as indicated in the figure and table legends. Differences between the two groups were analyzed by an unpaired Student’s *t*-test after testing the data for normal distribution and equal variance. Values are shown as mean  ±  standard error of the mean (SEM). Statistical significance was accepted for *p* < 0.05.

## 3. Results

### 3.1. Intravital Fluorescent Microscopic Analyses in the Dorsal Skinfold Chamber

**Figure 2:**
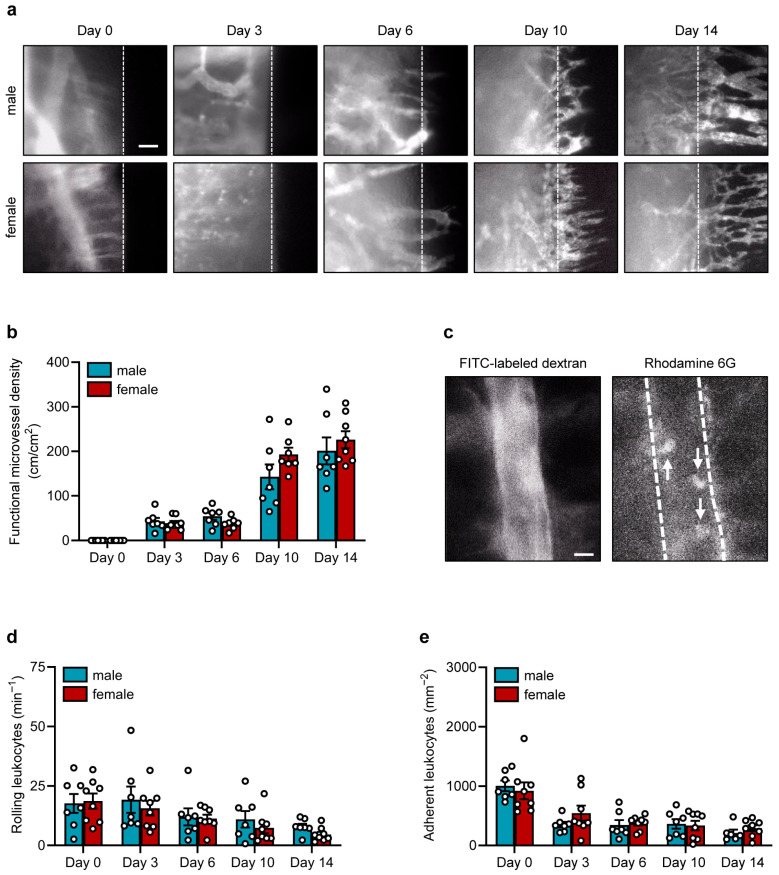
Intravital fluorescent microscopic analyses in the dorsal skinfold chamber. (**a**) Intravital fluorescent microscopic images of implanted polypropylene sutures in the dorsal skinfold chamber of male and female mice on days 0, 3, 6, 10 and 14. FITC-labeled dextran 150,000 was used for the visualization of blood-perfused microvessels. The borders of the implants are marked by broken lines. Scale bar: 30 μm. (**b**) Functional microvessel density (cm/cm^2^) in the granulation tissue surrounding the implanted polypropylene suture in the dorsal skinfold chambers of male and female mice on day 0, 3, 6, 10 and 14. Mean ± SEM (n = 7–8 animals each); no significant differences. (**c**) Intravital fluorescent microscopic images of a postcapillary venule (broken lines) on day 10 after implantation of a polypropylene suture into the dorsal skinfold chamber of a female mouse. FITC-labeled dextran 150,000 was used for visualization of blood-perfused microvessels (left panel). Rhodamine 6G was used for in situ staining of leukocytes (arrows; right panel). Scale bar: 10 µm. (**d**,**e**) Rolling leukocytes (min^−1^) (**d**) and adherent leukocytes (mm^−2^) (**e**) within postcapillary and collecting venules on day 0, 3, 6, 10 and 14 after implantation of polypropylene sutures into the dorsal skinfold chambers of male and female mice. Mean ± SEM (n = 7–8 animals each); no significant differences.

By means of the dorsal skinfold chamber in combination with the technique of intravital fluorescence microscopy, we first analyzed blood vessel formation and leukocyte-endothelial cell interactions after implantation of polypropylene sutures in male and female mice (Figure 2a–e).

Our in vivo microscopic analyses revealed the development of blood-perfused microvessels along the borders of the implanted sutures, which already started on day 3 after suture implantation (Figure 2a,b). The density of these vessels progressively increased over time and did not significantly differ between the groups of male and female mice (Figure 2a,b).

Moreover, we repeatedly assessed the interactions of individual leukocytes with the endothelium of postcapillary and collecting venules next to the polypropylene sutures (Figure 2c). For this purpose, we first measured the diameter, centerline RBC velocity, volumetric blood flow and wall shear rate of these vessels (Table 1).

Of note, we did not detect significant differences in these microhemodynamic parameters between male and female mice. We further did not detect sex-specific differences in the numbers of rolling and adherent leukocytes over time (Figure 2d,e). However, the number of adherent leukocytes was rather high on day 0 in both groups. After the last microscopy on day 14, blood samples of the animals were collected, and hematocrit (HCT), hemoglobin (HGB), and cellular composition were analyzed. This analysis also revealed comparable values between male and female mice (Table 2).

### 3.2. Immunohistochemical Analyses in the Dorsal Skinfold Chamber

**Figure 3:**
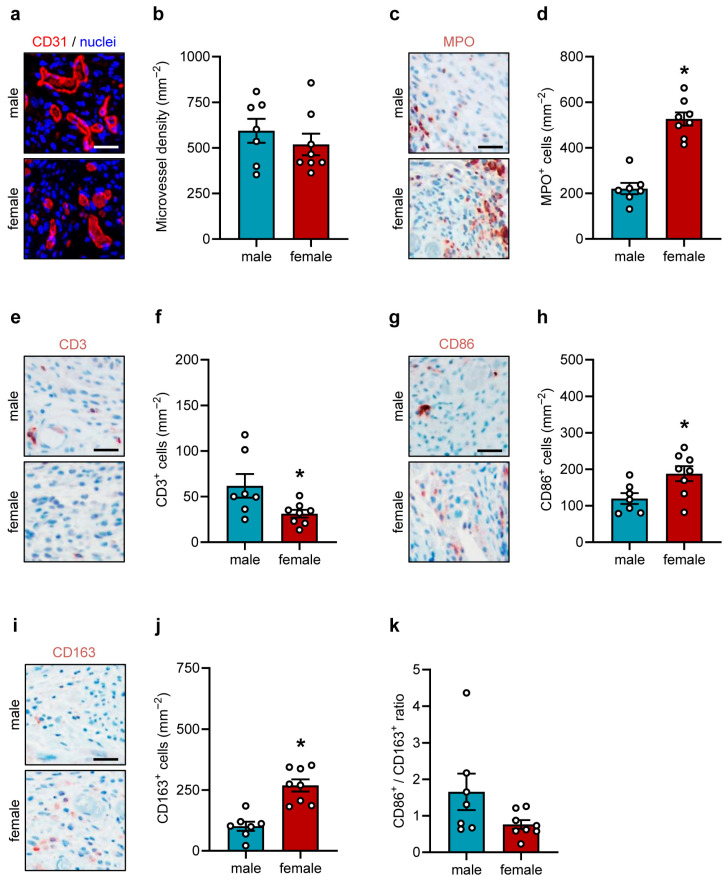
Immunohistochemical analyses in the dorsal skinfold chamber. (**a**) Representative immunohistochemical sections showing CD31^+^ microvessels in the granulation tissue surrounding the implanted polypropylene sutures in the dorsal skinfold chambers of male and female mice on day 14. Scale bar: 50 µm. (**b**) Microvessel density (mm^−2^) in the granulation tissue surrounding the implanted polypropylene sutures in the dorsal skinfold chambers of male and female mice on day 14. Mean ± SEM (n = 7–8 animals each); no significant differences. (**c**,**e**,**g**,**i**) Representative immunohistochemical sections showing MPO^+^ granulocytes (**c**), CD3^+^ lymphocytes (**e**), CD86^+^ (M1) macrophages (**g**), and C163^+^ (M2) macrophages (**i**) in the granulation tissue surrounding the implanted polypropylene sutures in the dorsal skinfold chambers of male and female mice on day 14. Scale bar: 50 µm. (**d**,**f**,**h**,**j**) MPO^+^ granulocytes (mm^−2^) (**d**), CD3^+^ lymphocytes (mm^−2^) (**f**), CD86^+^ (M1) macrophages (mm^−2^) (**h**) and C163^+^ (M2) macrophages (mm^−2^) (**j**) in the granulation tissue surrounding the implanted polypropylene sutures in the dorsal skinfold chambers of male and female mice on day 14. Mean ± SEM (n = 7–8 animals each). * *p* < 0.05 vs. male. (**k**) CD86^+^/CD163^+^ ratio in the granulation tissue surrounding the implanted polypropylene sutures in the dorsal skinfold chambers of male and female mice on day 14. Mean ± SEM (n = 7–8 animals each); no significant differences.

**Figure 4:**
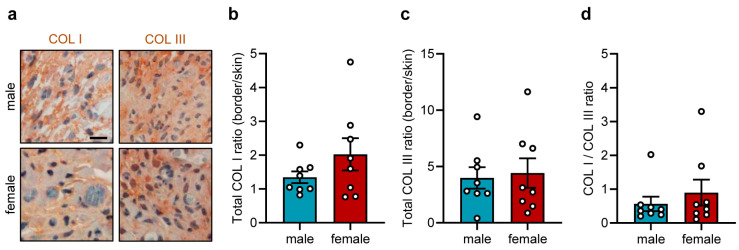
Tissue integration of implants in the dorsal skinfold chamber. (**a**) Representative immunohistochemical sections showing COL I (**left panel**) and COL III (**right panel**) in the granulation tissue surrounding the implanted polypropylene suture in the dorsal skinfold chambers of male and female mice on day 14. Scale bar: 25 µm. (**b**,**c**) Total COL I (**b**) and COL III ratio (border/skin) (**c**) in the granulation tissue surrounding the implanted polypropylene suture in the dorsal skinfold chambers of male and female mice on day 14. Mean ± SEM (n = 8 animals each); no significant differences. (**d**) COL I/COL III ratio in the granulation tissue surrounding the implanted polypropylene suture in the dorsal skinfold chambers of male and female mice on day 14. Mean ± SEM (n = 8 animals each); no significant differences.

After the in vivo experiments, the border zones of the implanted sutures were further immunohistochemically analyzed (Figure 3a–k and Figure 4a–d).

CD31 staining demonstrated a comparably high microvessel density in both groups (Figure 3a,b). Moreover, we evaluated the infiltration of immune cells in the border zones of the implants by means of immunohistochemical sections stained against MPO, CD3, CD86 and CD163 (Figure 3c–k). These analyses showed that the number of MPO^+^ neutrophilic granulocytes was significantly higher in females, whereas the number of CD3^+^ T-lymphocytes was reduced when compared to males (Figure 3c–f). CD86^+^ (M1) macrophages and CD163^+^ (M2) macrophages were significantly increased in females (Figure 3g–j). However, the M1/M2 ratio was similar between both groups (Figure 3k).

To further investigate the integration of the sutures into the host tissue, the COL content of the suture border zones was quantified while differentiating between COL I and COL III. These analyses showed comparable amounts of COL I and COL III around the implanted sutures in male and female mice (Figure 4a–c), resulting in a COL I/COL III ratio of 0.5–0.9 (Figure 4d).

### 3.3. Immunohistochemical Analyses in the Subcutaneous Flank Model

**Figure 5:**
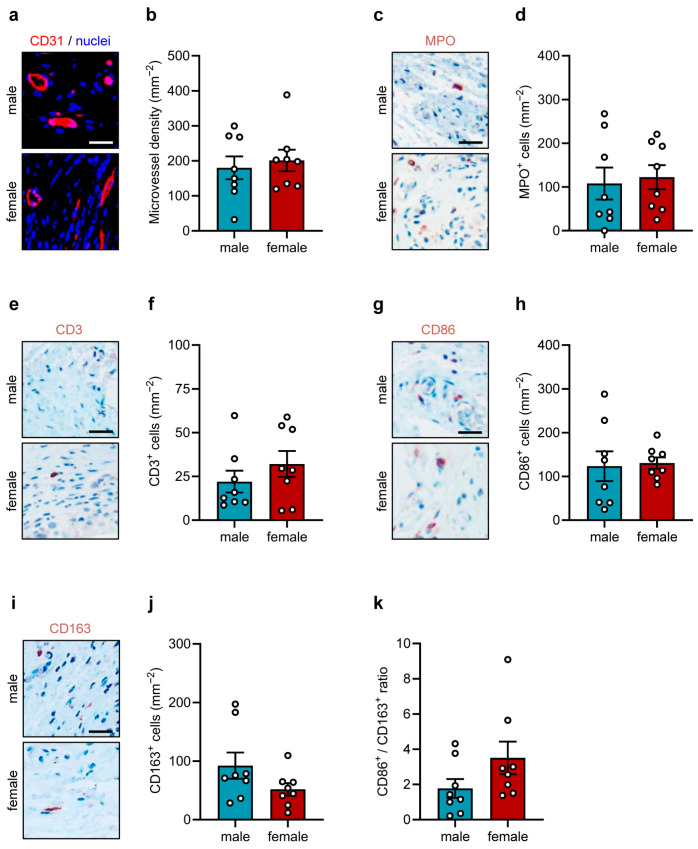
Immunohistochemical analyses in the subcutaneous flank model. (**a**) Representative immunohistochemical sections showing CD31^+^ microvessels in the granulation tissue surrounding the implanted polypropylene suture within the flanks of male and female mice on day 28. Scale bar: 50 µm. (**b**) Microvessel density (mm^−2^) in the granulation tissue surrounding the implanted polypropylene suture within the flanks of male and female mice on day 28. Mean ± SEM (n = 8 animals each); no significant differences. (**c**,**e**,**g**,**i**) Representative immunohistochemical sections showing MPO^+^ granulocytes (**c**), CD3^+^ lymphocytes (**e**), CD86^+^ (M1) macrophages (**g**) and C163^+^ (M2) macrophages (**i**) in the granulation tissue surrounding the implanted polypropylene suture within the flanks of male and female mice on day 28. Scale bar: 50 µm. (**d**,**f**,**h**,**j**) MPO^+^ granulocytes (mm^−2^) (**d**), CD3^+^ lymphocytes (mm^−2^) (**f**), CD86^+^ (M1) macrophages (mm^−2^) (**h**) and C163^+^ (M2) macrophages (mm^−2^) (**j**) in the granulation tissue surrounding the implanted polypropylene suture within the flanks of male and female mice on day 28. Mean ± SEM (n = 8 animals each); no significant differences. (**k**) CD86^+^/CD163^+^ ratio in the granulation tissue surrounding the implanted polypropylene suture within the flanks of male and female mice on day 28. Mean ± SEM (n = 8 animals each); no significant differences.

Using the subcutaneous flank model, we analyzed the chronic foreign body response to implanted polypropylene sutures after an implantation period of 28 days by means of immunohistochemistry (Figure 5a-k). Immunohistochemical detection of CD31^+^ microvessels showed no significant differences in microvessel density between male and female mice (Figure 5a,b). However, when compared to the results in the dorsal skinfold chamber model, the microvessel density was 2.9-fold lower (compare Figure 3a,b with Figure 5a,b). Additional analyses revealed that the sex of the animals affected neither the numbers of MPO^+^ granulocytes nor CD3^+^ T-lymphocytes (Figure 5c–f). The numbers of CD86^+^ (M1) and CD163^+^ (M2) macrophages also did not differ between the two groups (Figure 5g–j), resulting in a comparable M1/M2 ratio (Figure 5k).

**Figure 6:**
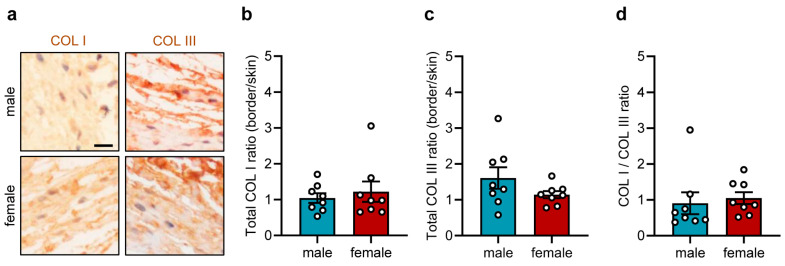
Tissue integration of implants in the subcutaneous flank model. (**a**) Representative immunohistochemical sections showing COL I (**left panel**) and COL III (**right panel**) in the granulation tissue surrounding the implanted polypropylene suture within the flanks of male and female mice on day 28. Scale bar: 25 µm. (**b**,**c**) Total COL I (**b**) and COL III ratio (border/skin) (**c**) in the granulation tissue surrounding the implanted polypropylene suture within the flanks of male and female mice on day 28. Mean ± SEM (n = 8 animals each); no significant differences. (**d**) COL I/COL III ratio in the granulation tissue surrounding the implanted polypropylene suture within the flanks of male and female mice on day 28. Mean ± SEM (n = 8 animals each); no significant differences.

Finally, analysis of COL I and COL III-stained sections showed similar tissue integration of the implanted sutures in both groups (Figure 6a–c). The COL I/COL III ratio was ~1 in male and female mice (Figure 6d).

## 4. Discussion

Sutures are essential medical biomaterials; however, their implantation triggers angiogenic and immunological host reactions that may influence healing outcomes [21]. While polypropylene sutures are widely applied across surgical disciplines, the biological response to this suture material may vary depending on patient-related factors such as sex [22,23,24]. Therefore, we herein examined the acute and chronic foreign body response to polypropylene sutures implanted in male and female mice.

The dorsal skinfold chamber model has previously been used to study the vascularization of transplanted tissues, including pancreatic islets [25], and implanted biomaterials, such as scaffolds for tissue engineering [26]. In the present study, polypropylene sutures were freely placed onto the striated muscle tissue within dorsal skinfold chambers of male and female recipients. This allowed repeated assessment of the sex-dependent angiogenic and inflammatory host tissue response to the implants throughout an observation period of 14 days by means of intravital fluorescence microscopy.

The in vivo analyses demonstrated early vascularization along the borders of the implanted polypropylene sutures in male and female mice. Moreover, the functional microvessel density progressively increased over time, reflecting continuous angiogenic activity of the microvasculature induced by the implanted material. Additional immunohistochemical staining supported these findings. Of note, the ~2.5-fold lower microvessel density observed in the chronic flank model suggests that the initially dense microvascular network underwent structural remodeling over time, leading to a well-organized microvascular architecture around the implanted suture after a longer observation period of 4 weeks. However, direct comparison of data from the dorsal skinfold chamber model and the subcutaneous flank model should be taken with caution, as the two models represent two different experimental systems with varying microenvironments. Therefore, comparisons between these two models should be interpreted as being solely descriptive and not in a quantitative manner.

Furthermore, intravital fluorescence microscopy revealed no sex-specific differences in microhemodynamic parameters, indicating that both male and female animals exhibited comparable perfusion conditions within postcapillary and collecting venules. Leukocyte-endothelial cell interactions in these vessels were also comparable between male and female mice throughout the entire observation period. Of interest, the number of adherent leukocytes was rather high on day 0 in both groups. This observation may be explained by acute tissue manipulation during suture implantation.

Additional immunohistochemical analyses revealed sex-dependent differences in specific immune cell subpopulations, with significantly higher numbers of MPO^+^ neutrophilic granulocytes and lower numbers of CD3^+^ T-lymphocytes in the granulation tissue surrounding the implanted sutures in female mice when compared to males. This is in line with previous studies reporting that females exhibit an overall stronger innate immune activation [8,27]. Neutrophilic granulocytes represent the first inflammatory cell type to infiltrate a wound site, where they not only perform phagocytosis but also attract macrophages by releasing signaling molecules, such as interleukin-1β [28,29]. In line with this finding, females exhibited significantly higher fractions of both CD86^+^ pro-inflammatory (M1) and CD163^+^ anti-inflammatory (M2) macrophages in the present study. Accordingly, the M1/M2 ratio was similar between male and female mice. This indicates that although sex determines the extent of the acute inflammatory response, it does not alter the subsequent polarization state of macrophages. The latter result may also explain why we did not detect sex-specific differences in the chronic foreign body response to the sutures 4 weeks after implantation into the flanks of male and female mice. In fact, macrophage polarization is a key determinant of biomaterial integration because a balanced M1/M2 ratio promotes controlled inflammation and facilitates the transition towards constructive tissue remodeling [9,28]. Accordingly, the COL I/COL III ratio in the granulation tissue was also comparable in male and female mice.

Finally, it should be mentioned that this study also faces some limitations. First, although our immunohistochemical analyses on day 14 suggest a stronger early innate immune response in females, these findings were not confirmed by our intravital microscopic analyses or by the results from the chronic subcutaneous flank model. Therefore, the observed sex-specific differences appear to be restricted to specific cell populations, i.e., neutrophilic granulocytes and macrophages, at a single time point and do not translate into detectable long-term effects. Second, while we propose that these differences may be related to enhanced immune responses in females, this hypothesis has not been directly investigated in the present study. Accordingly, it would be of interest to further analyze the underlying molecular mechanisms, such as cytokine expression and signaling pathways, in females when compared to males. Additionally, the potential influence of the estrous cycle on the foreign body reaction in female mice should be considered in future studies. Moreover, it may be speculated that sex-specific effects differ markedly depending on the severity of a foreign body response. Hence, we suggest that sex-specific differences in response to other types of suture materials, such as degradable polycaprolactone or polylactic acid, should also be analyzed.

## 5. Conclusions

This study provides, for the first time, a comprehensive analysis of sex-specific differences in vascularization, immune cell infiltration, and tissue integration following implantation of polypropylene sutures. By combining the acute dorsal skinfold chamber with the chronic subcutaneous flank model, we could demonstrate that females exhibit a stronger early innate immune response to implanted sutures. Given the growing recognition of sex as a fundamental biological variable in the life sciences [23], our results highlight the importance of including both sexes in the preclinical evaluation of biomaterials to ensure the generalizability of biocompatibility and biomaterial performance across diverse patient populations in clinical practice.

## Figures and Tables

**Table 1:** Diameter (µm), centerline RBC velocity (µm/s), volumetric blood flow (pL/s), and wall shear rate (s^−1^) of postcapillary and collecting venules on days 0, 3, 6, 10 and 14 after implantation of polypropylene sutures into the dorsal skinfold chambers of male and female mice.

	Day 0	Day 3	Day 6	Day 10	Day 14
Diameter (µm):					
Male	31.2 ± 1.3	26.7 ± 2.3	24.5 ± 2.8	22.6 ± 2.3	16.8 ± 0.9
Female	27.9 ± 1.8	23.6 ± 1.3	21.5 ± 1.1	22.5 ± 3.7	20.3 ± 2.9
Centerline RBC velocity (µm/s):					
Male	229.7 ± 26.1	244.0 ± 26.2	278.8 ± 37.1	182.1 ± 15.4	208.3 ± 17.6
Female	228.1 ± 28.1	241.6 ± 16.3	227.7 ± 20.9	214.3 ± 15.1	240.8 ± 37.1
Volumetric blood flow (pL/s):					
Male	107.3 ± 10.9	92.9 ± 19.9	108.5 ± 39.9	53.5 ± 14.7	30.1 ± 4.3
Female	88.9 ± 13.9	67.4 ± 8.2	55.1 ± 9.9	64.0 ± 22.2	46.8 ± 8.8
Wall shear rate (s^−1^):					
Male	60.9 ± 8.9	76.7 ± 10.3	92.1 ± 8.0	67.5 ± 6.9	100.5 ± 9.3
Female	67.5 ± 9.5	83.7 ± 7.0	84.8 ± 5.5	86.6 ± 10.3	107.1 ± 17.4

Mean ± SEM (n = 7–8 animals each); no significant differences.

**Table 2:** Cellular composition of blood from male and female mice on day 14 after implantation of polypropylene sutures into the dorsal skinfold chamber.

	Male	Female
HCT (%)	41.4 ± 4.0	48.9 ± 2.2
HGB (g/dL)	14.3 ± 0.3	14.3 ± 0.3
Erythrocytes (×10^12^/L)	8.4 ± 0.8	9.4 ± 0.5
Leukocytes (×10^9^/L)	7.0 ± 1.3	8.3 ± 1.2
Neutrophils (×10^9^/L)	0.7 ± 0.1	1.0 ± 0.2
Lymphocytes (×10^9^/L)	6.1 ± 1.1	7.1 ± 1.0
Monocytes (×10^9^/L)	0.2 ± 0.1	0.2 ± 0.1
Platelets (×10^9^/L)	360.1 ± 87.5	527.9 ± 68.2

Mean ± SEM (n = 8 animals each); no significant differences.

## Data Availability

The original contributions presented in this study are included in the article. Further inquiries can be directed to the corresponding author.

## Abbreviations

The following abbreviations are used in this manuscript:

FITC	Fluorescein isothiocyanate
MPO	Myeloperoxidase
COL	Collagen
NIH	National Institutes of Health
i.p.	Intraperitoneal
ROIs	Regions of interest
RBC	Red blood cell
PFA	Paraformaldehyde
SEM	Standard error of the mean
HCT	Hematocrit
HGB	Hemoglobin
